# Supplementary material for: Ethanol exposure increases mutation rate through error-prone polymerases
Source: Nat Commun. 2020 Jul 21;11:3664. doi: 10.1038/s41467-020-17447-3 (PMC7374746; doi:10.1038/s41467-020-17447-3)
Supplement: Supplementary file 1 — Supplementary Information [file 41467_2020_17447_MOESM1_ESM.pdf]

Supplementary information

Ethanol exposure increases mutation rate through error-prone polymerases.

Voordeckers *et al.*

Supplementary Methods

RNA sequencing analysis

Reads were analysed by a reference-based approach using the Tuxedo pipeline, based on the protocol laid out in the work of Trapnel and coworkers[1,2]. Raw reads were aligned to the *S. cerevisiae* reference genome (release 64) using the Tophat aligner v2.0.13[3] and transcript abundances were estimated using the cufflinks v2.2.1[4]. Identification of genes showing differential expression between samples was performed with cuffdiff v2.2.1[1], with cross-replication dispersion addressed by pooling the duplicate reads for each sample and running cuffdiff in a pooled mode. Term enrichment analysis for lists of genes found to be differentially expressed was performed with DAVID 6.7[5-7] using a cutoff of 1.3, as recommended by the authors.

Yeast live cell imaging and fluorescence

Cells for Figure 6 were grown as described in[8] and Methods section. Fluorophores used in Figure 6 were cyan fluorescent protein (CFP, clone W7)[9], yellow fluorescent protein (YFP, clone 10C)[10] and red fluorescent protein (RFP, clone yEmRFP; or mCherry)[11]. Fluorophores were visualized on a Deltavision Elite microscope (Applied Precision, Inc) equipped with a 100x objective lens (Olympus U-PLAN S-APO, NA 1.4), a cooled Evolve 512 EMCCD camera (Photometrics, Japan), and an Insight solid-state illumination source (Applied Precision, Inc). Images were acquired using softWoRx software version 7.0.0 (Applied Precision, Inc) software and fluorescence intensities measured with Volocity software version 5.4 (PerkinElmer).

Replication Fork Speed Measurements

Replication fork progression was quantitatively analyzed following 50 µM 5-ethynyl-2'-deoxyuridine (EdU) incorporation and DNA fiber combing, following the protocol from[12]. Briefly, cells were grown overnight at 25 °C to a density of 5 × 10^6 cells/ml in 100 ml of complete synthetic medium. BrdU was added to the medium to a final concentration of 40 µg/ml. Cells were arrested after 90 min with 0.1% sodium azide, collected by centrifugation and resuspended in 10 mM Tris–HCl, 50 mM EDTA, pH 8.0 at 4 °C. Genomic DNA was prepared in low melting point (LMP) agarose plugs. To this end, cells were washed with 10 mM of TE_{50} buffer and cell concentration was determined with a cell counter. Cells were resuspended in prewarmed Zymolyase buffer (42 °C) to a final concentration of 1 × 10^9 cells/ml and carefully mixed with an equal volume of molten LMP agarose (42 °C). The cellular suspension was transferred into a plug mold sealed with tape to generate 90 µl plugs containing 5 × 10^7 cells or approximately 850 ng of genomic DNA per plug. Solidified plugs were transferred into 12 ml round-bottom polypropylene tubes using a rubber bulb and were incubated overnight at 37 °C

in Zymolyase buffer (2 ml for 5 plugs) to digest the cell wall. Next, plugs are incubated for 48 h at 42 °C in Proteinase K buffer (2 ml for 5 plugs) and were extensively washed (5 × 10 min) in 10 ml TE_{50} buffer. For each condition, one genomic DNA plug was transferred into a round-bottom polycarbonate tube containing 1.5 µl YOYO-1 in 100 µl TE_{50} buffer and incubated for 30 min in the dark to stain genomic DNA. Then, plugs were washed 3 × 5 min with 10 ml of TE buffer and incubated for 5 min in 5 ml of 50 mM MES buffer at pH 5.7. The MES buffer was replaced with 5 ml of fresh buffer and the polycarbonate tube was transferred to a heating block set at 65 °C until complete melting of agarose plugs. Agarose polymers were digested overnight at 42 °C by addition of 3 units of β-agarase. The following day, the DNA solution was incubated again for 10 min at 65 °C and stored at room temperature until use. DNA combing was performed on silanized coverslips. The DNA solution was carefully transferred into a 2–3 ml Teflon reservoir and a silanized coverslip was incubated into the DNA solution for 5 min at room temperature. Coverslip were carefully removed from the reservoir at a constant speed of 250 µm/s, using the DNA combing system distributed by Genomic Vision. The density of DNA fibers was determined by visual inspection with a microscope, using an oil-immersion 40× objective and a FITC filter block. Coverslips are baked for 2 h at 60 °C to crosslink DNA fibers to the glass surface. Coverslips were attached to glass slides with cyanoacrylate glue, labeled with a diamond tip engraving pen or a solvent resistant pen and stored at −20 °C until use. DNA fibers attached to coverslips were dehydrated by incubating slides for 5 min in Coplin jars containing 70%, 90% and 100% ethanol. Slides were then incubated for 22–25 min in 1 M NaOH to denature the DNA duplex and are washed extensively with PBS pH 7.4 to neutralize NaOH (at least five washes of 1 min each). Slides were saturated for 15 min in PBS/T buffer containing 1% BSA. Primary antibodies were diluted in 20 µl of PBS/T buffer and were added directly on the slide. A second coverslip was used to limit evaporation. Slides were incubated for 45 min at 37 °C in a humid chamber and were washed for 5 × 2 min with PBS/T before the addition of secondary antibodies. After a second incubation for 30 min at 37 °C, slides were washed for 5 × 2 min with PBS/T buffer, air dried and mounted with 18 µl of Prolong Gold antifade reagent (Molecular Probes). EdU replication tracks were detected using Alexa Fluor 555 Azide and Click chemistry, using 20 µL of the following mix per coverslip: 16.7 µL of H_2O, 0.8 µL of 100 mM Copper sulfate, 0.5 µL of 0.5 mg/mL Alexa Fluor® 555 Azide (Molecular Probes A20012) and 2 µL of 100 mM Sodium ascorbate.

DNA fibers were detected using mouse anti-ssDNA (DSHB, http://dshb.biology.uiowa.edu/autoimmune-ssDNA;)[82] and goat anti-mouse coupled to Alexa Fluor 647 (ThermoFisher, A21241). Antibodies were used in a 1/50 dilution. 300 to 600 individual EdU tracks were counted for each experimental condition. Statistical analyses were performed with GraphPad Prism 7.

Supplementary Information Figures and Tables

Source data for Supplementary figures are provided as a Source Data file.

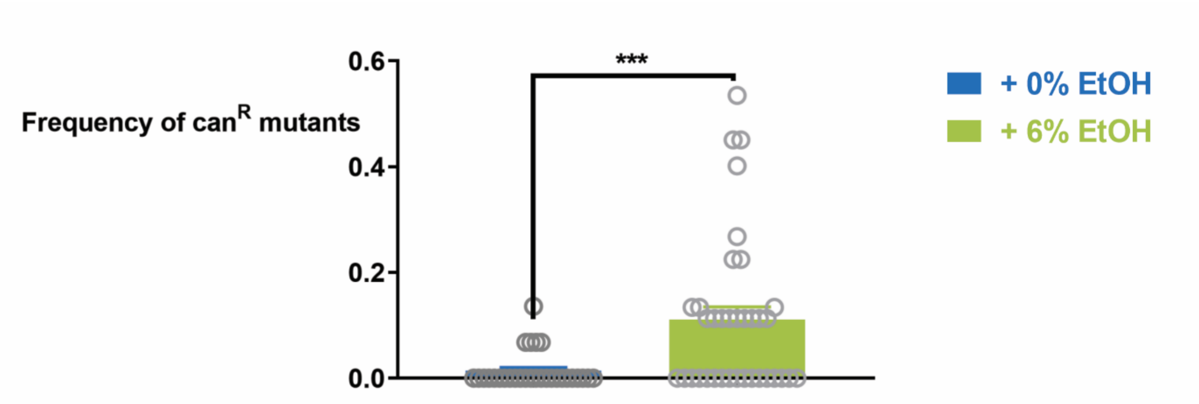

Supplementary Figure 1. Ethanol increases can^R mutant frequency in W303.

Cells of strain VK3847 were exposed to 0 or 6% (v/v) ethanol for 2h, and subsequently plated on canavanine plates. Bars represent average of at least 29 biological replicates +/- SEM. N= 29 and 26 for 0 and 6% ethanol, respectively. Statistical significance was assessed using a two-tailed unpaired t-test with Welch's correction, $***P < 0.001$. Specifically, p value = 0.0005.

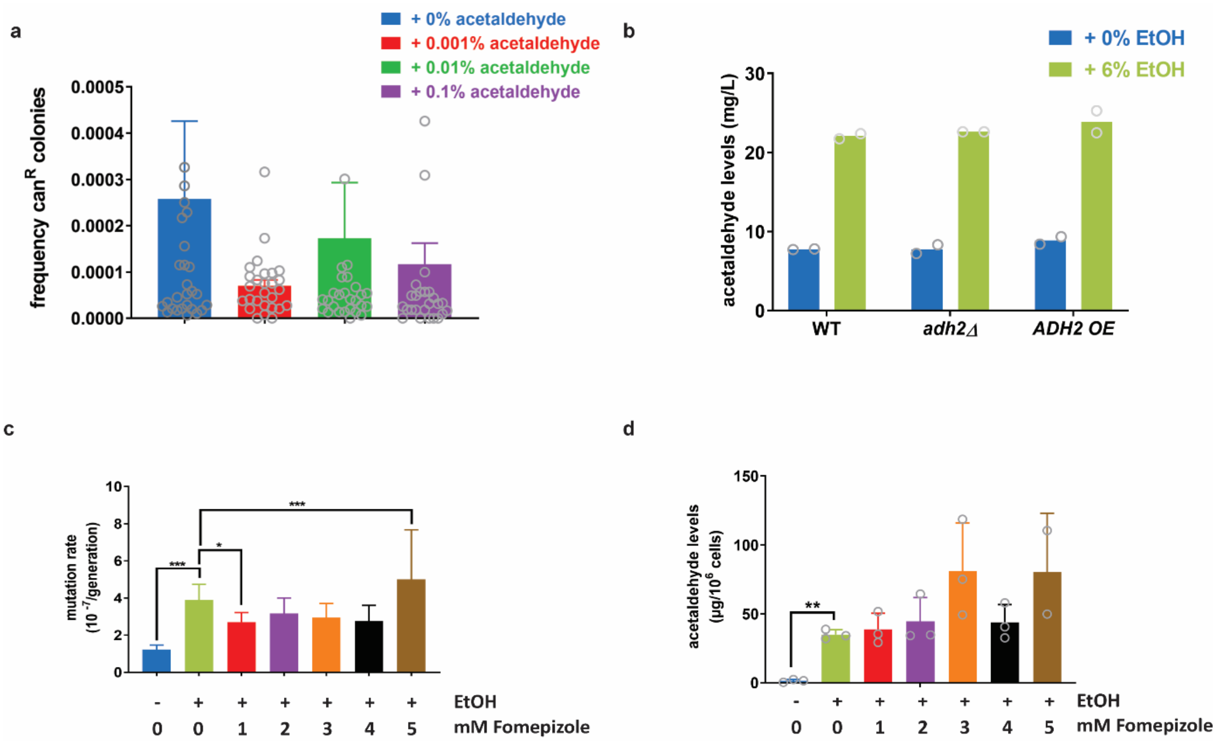

Supplementary Figure 2. Additional experiments performed to investigate the role of acetaldehyde in the mutagenic effect of ethanol.

a. **Extracellular addition of acetaldehyde does not alter *can^R* frequency.**
Cells (VK111) in synthetic media (2% glucose), were exposed to the indicated acetaldehyde concentrations (v/v), see also methods. Data represents average of at least 18 biological replicates +/- SEM. N= 18 and 27, for 0% acetaldehyde and all other conditions, respectively. Statistical significance was assessed using a two-tailed unpaired t-test with Welch's correction. There is no statistically significant difference between the 0% acetaldehyde condition and any of the other acetaldehyde conditions.

b. **Altered *ADH2* levels do not change intracellular acetaldehyde levels.**
Intracellular acetaldehyde was measured by HPLC in strains VK111, EV14 and EV19. Bars represent average of two biological replicates.

c. **Fomepizole addition does not abolish mutagenic effect of ethanol.**
Cells (VK111) were grown in synthetic media (2% glucose) with 0 or 6% EtOH and the indicated fomepizole concentrations (v/v). For each condition, 54 cultures were analyzed. Mutation rate estimates, as determined by fluctuation assays on canavanine, are shown as centre of error bars. Error bars represent 95% confidence intervals. Statistical significance of differences in mutation rates was assessed using a likelihood ratio test. *$P < 0.05$, ***$P < 0.001$. P-values can be found in Supplementary Table 1.

d. **Fomepizole addition does not alter acetaldehyde levels.**
Cells (VK111) were grown in synthetic media (2% glucose) with 0 or 6% ethanol and the indicated fomepizole concentrations. Bars represent average of three replicates per strain +/- SD. Statistical significance was assessed using a two-tailed unpaired t-test with Welch's correction. **$P < 0.01$. P-values can be found in Supplementary Table 1.

a

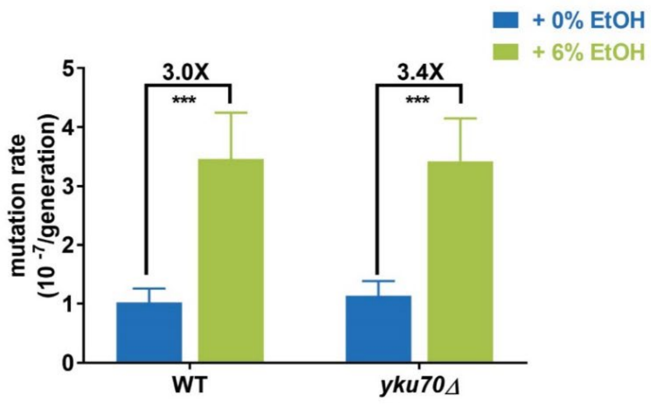

b

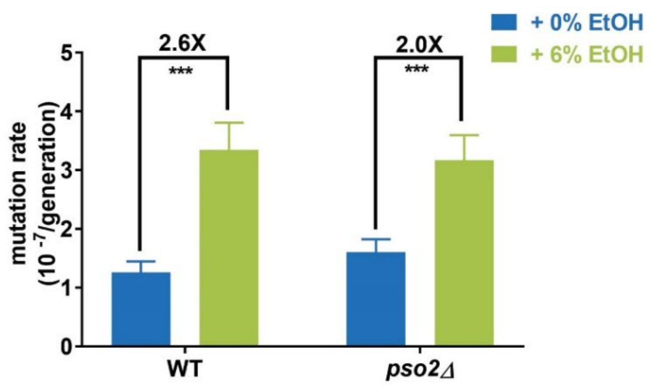

Supplementary Figure 3.

a. Mutagenic effect of ethanol does not depend on NHEJ.

Cells (VK111 and VK3918) were grown in synthetic media (2% glucose), supplemented with the indicated ethanol concentrations (v/v). For each condition, 54 cultures were analyzed. Mutation rate estimates, as determined by fluctuation assays on canavanine, are shown as centre of error bars. Error bars represent 95% confidence intervals. Statistical significance of differences in mutation rates was assessed using a likelihood ratio test. ***$P < 0.001$. Specifically, p= 4.479 10^{-13} and 2.044 10^{-12} for WT and *yku70*Δ respectively.

***b. PSO2* deletion does not abolish the mutagenic effect of ethanol.**

Cells (VK111 and VK3928) were grown in synthetic media (2% glucose), supplemented with the indicated ethanol concentrations (v/v). For each condition, 108 cultures were analyzed. Mutation rate estimates, as determined by fluctuation assays on canavanine, are shown as centre of error bars. Error bars represent 95% confidence intervals. Statistical significance of differences in mutation rates was assessed using a likelihood ratio test. ***$P < 0.001$. Specifically, p=0 and p=2.576 10^{-12} for WT and *pso2*Δ respectively.

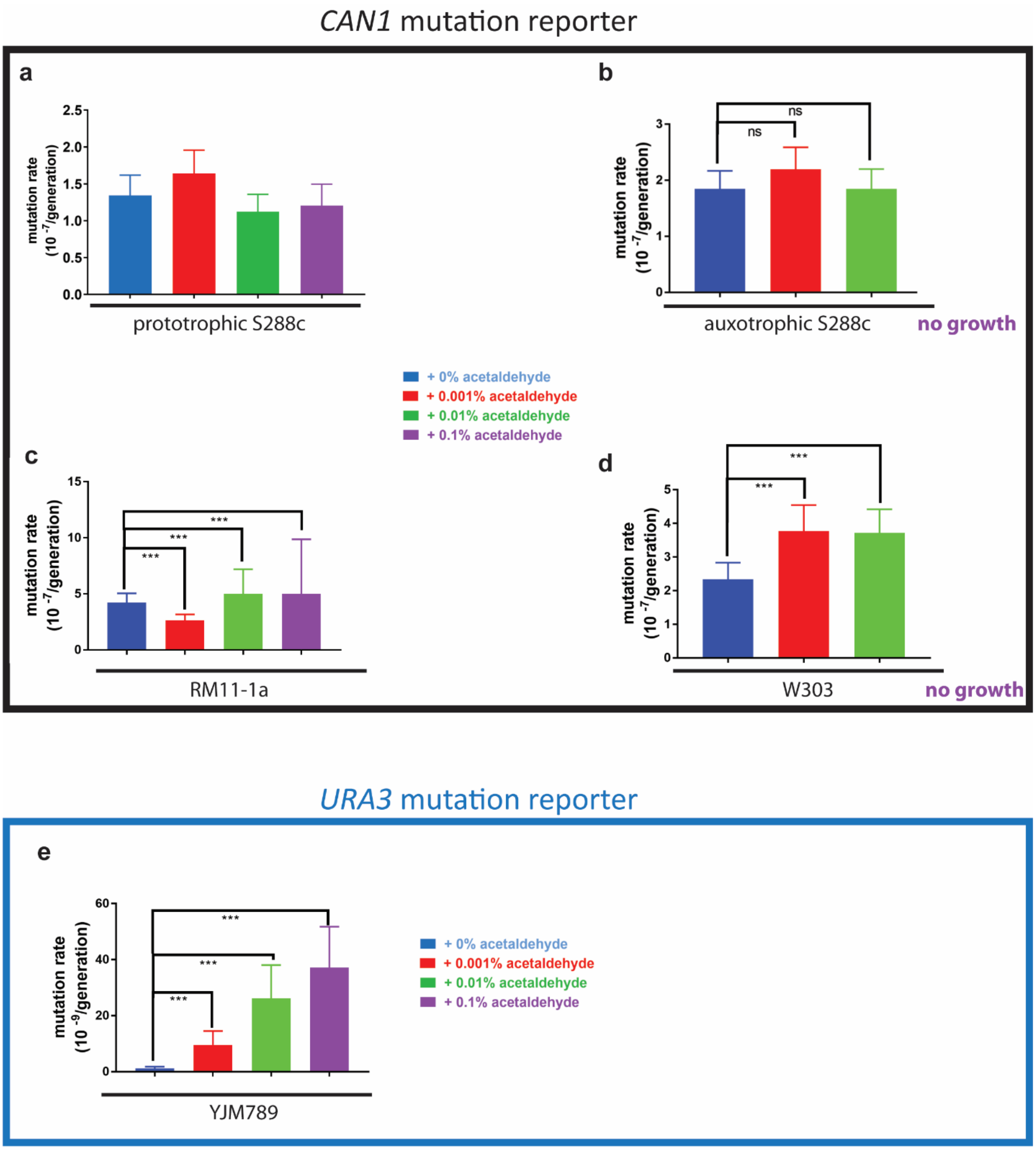

Supplementary Figure 4. Effect of acetaldehyde on mutation rate in different yeast strains.

Cultures of different yeast strains were grown in synthetic media (2% glucose) and indicated acetaldehyde concentrations (v/v). N= 41 and 54, for RM11-1a in 0.1 % acetaldehyde and all other strains and conditions, respectively. Mutation rate estimates, as determined by fluctuation assays on canavanine or FOA, are shown as centre of error bars. Error bars represent 95% confidence intervals. Statistical significance of differences in mutation rates was assessed using a likelihood ratio test. $*P < 0.05$, $***P < 0.001$. Specific p-values can be found in Supplementary Table 1.

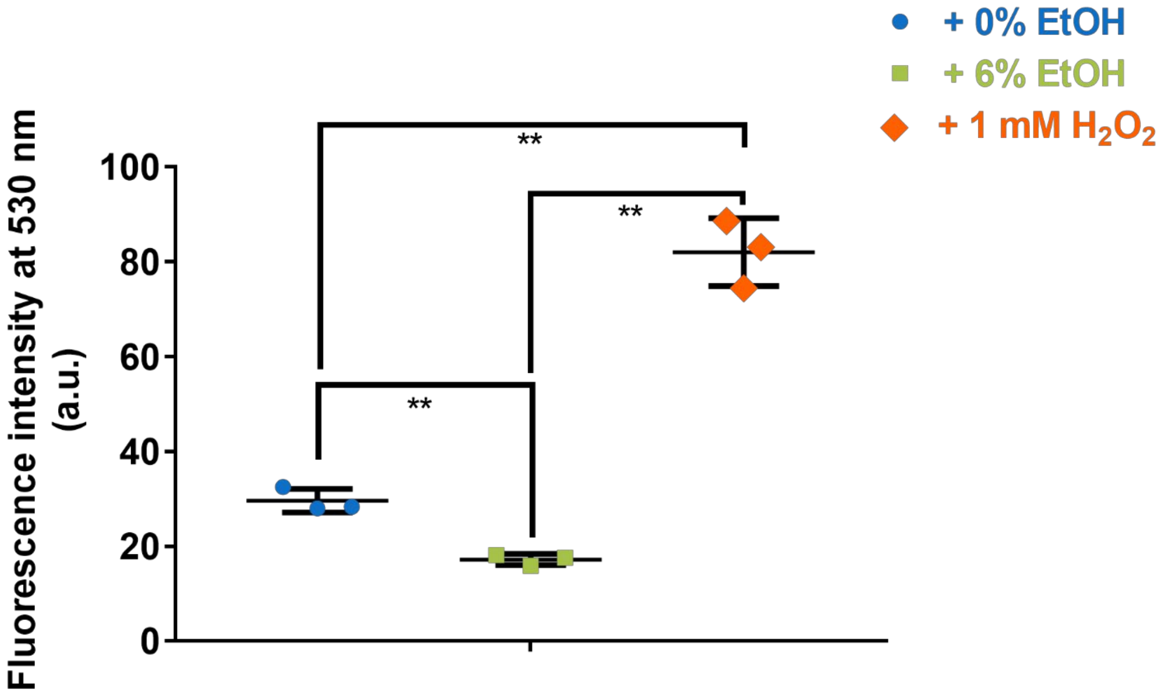

Supplementary Figure 5. Measurement of ethanol-induced oxidation.

Cells (VK111) were grown in synthetic medium (2% glucose) and incubated with the oxidant-sensitive probe H_2DCFDA and the indicated chemicals, after which fluorescence was analysed using flow cytometry. Three biological replicates of 50 000 cells were analysed per condition. Bars represent average +/- SD. Statistical significance was assessed using a two-tailed unpaired t-test with Welch's correction. **$P < 0.01$. Specifically, p-value= 0.0056 for 0-6% EtOH comparison, 0.0029 for 0- 1mM H_2O_2 and 0.0033 for 6%-1 mM H_2O_2.

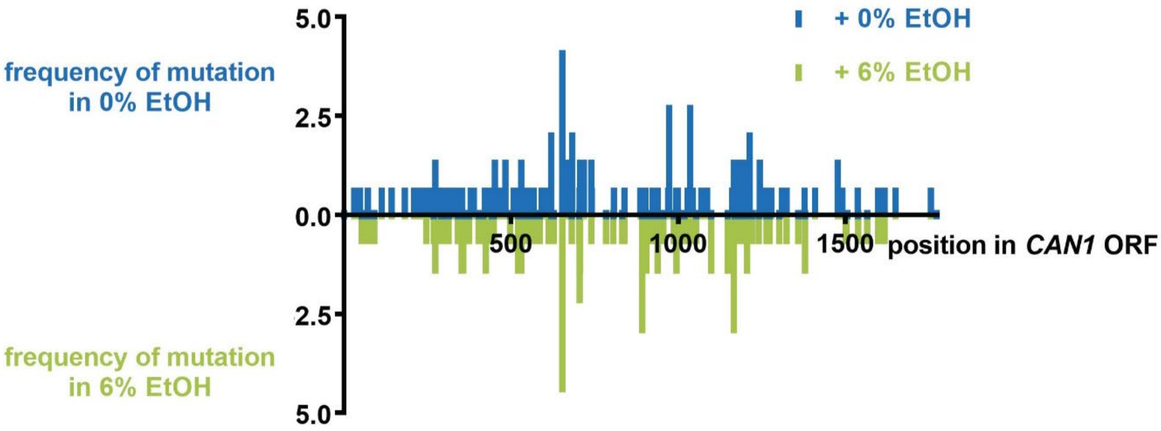

Supplementary Figure 6. Distribution of mutations identified in *CAN1* ORF.

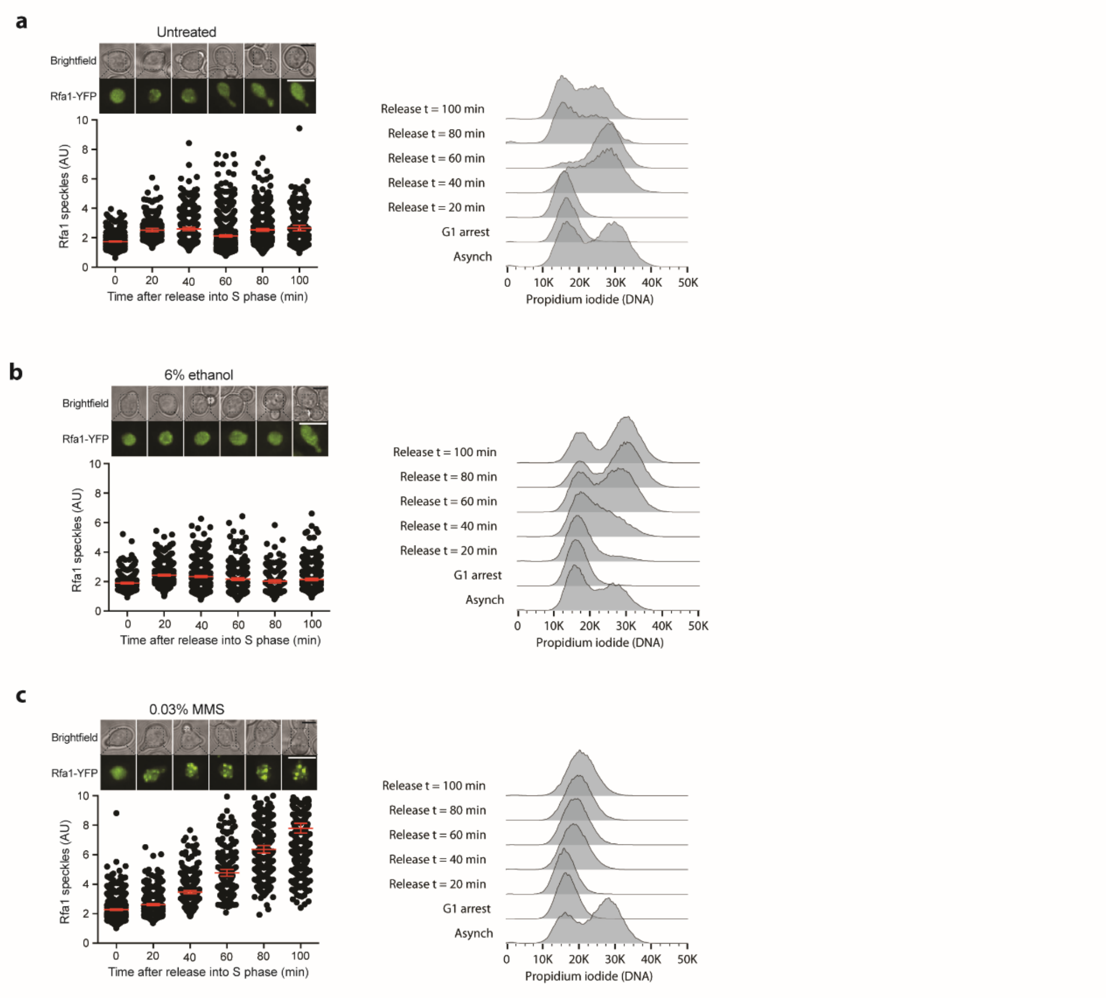

Supplementary Figure 7. Single-stranded DNA does not form extensively upon ethanol exposure. Wild-type (ML147-2B) cells were arrested in G1 by alpha-factor and released into S phase in the absence or presence of 6% ethanol or 0.03% MMS. Cells were analyzed for DNA content (right panels) and the presence of Rfa1-YFP speckles/foci as a measure for ssDNA at the indicated time points (left panels). Representative microscopy images are shown above each column in the graph. Scale bars, 3 μm. Red lines indicate average, error bars indicate 95% confidence intervals. For each time point, two biological replicates were analyzed. Specifically, for the Rfa speckles, N= 1023, 604, 332, 847, 686 and 384 for the different timepoints in 0% ethanol, N= 408, 610, 568, 344, 187, 372 for the different timepoints in 6% ethanol, and N= 705, 434, 318, 231, 345, 461 for the different timepoints in MMS.Y-axis truncated at the value 10 for better display.

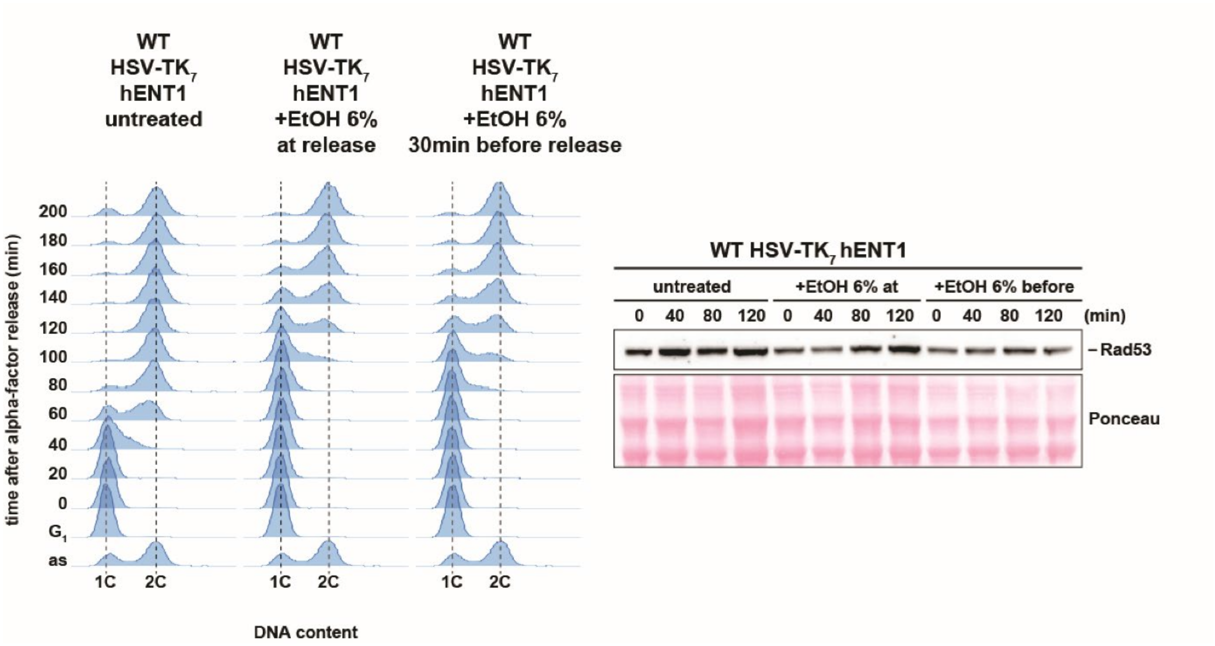

Supplementary Figure 8. Rad53 is not phosphorylated upon ethanol exposure.
Wild-type cells (PP2226) were synchronized in G_1 with α-factor prior to release with pronase in the presence of 6% ethanol. Cells were collected at the indicated times, and Rad53 phosphorylation was monitored by Western blot. This experiment has been repeated three times independently with similar results.

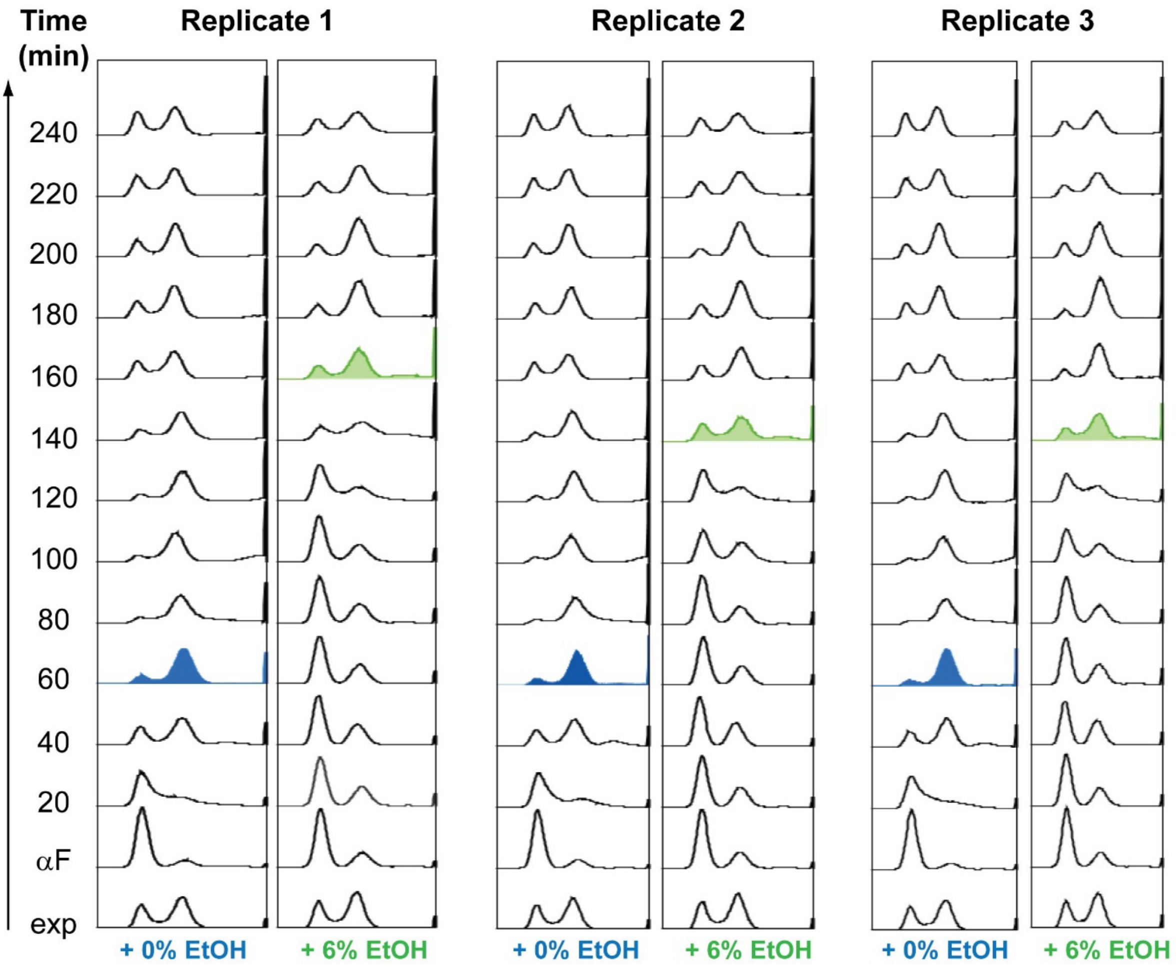

Supplementary Figure 9. Replication is slower in ethanol-exposed cells.

Ethanol stress causes a delay in cell cycle progression. DNA replication was assessed using flow cytometry. The colored plots indicate the timepoint at which populations have reached the 2C peak. Cell populations (VK91) in 0 and 6% ethanol post release from synchronization with mating factor are shown. A sample was taken every 20 min post-release, up to 240 min. DNA content of cells was determined by PI staining and samples were analyzed by flow cytometry. 50.000 events were counted for every sample. The 1C peak corresponds to cells in the G0/G1 phase. The 2C peak (double the amount of fluorescence intensity thus double the amount of DNA) corresponds to cells in the G2/M phase. This experiment was done for three biological replicates.

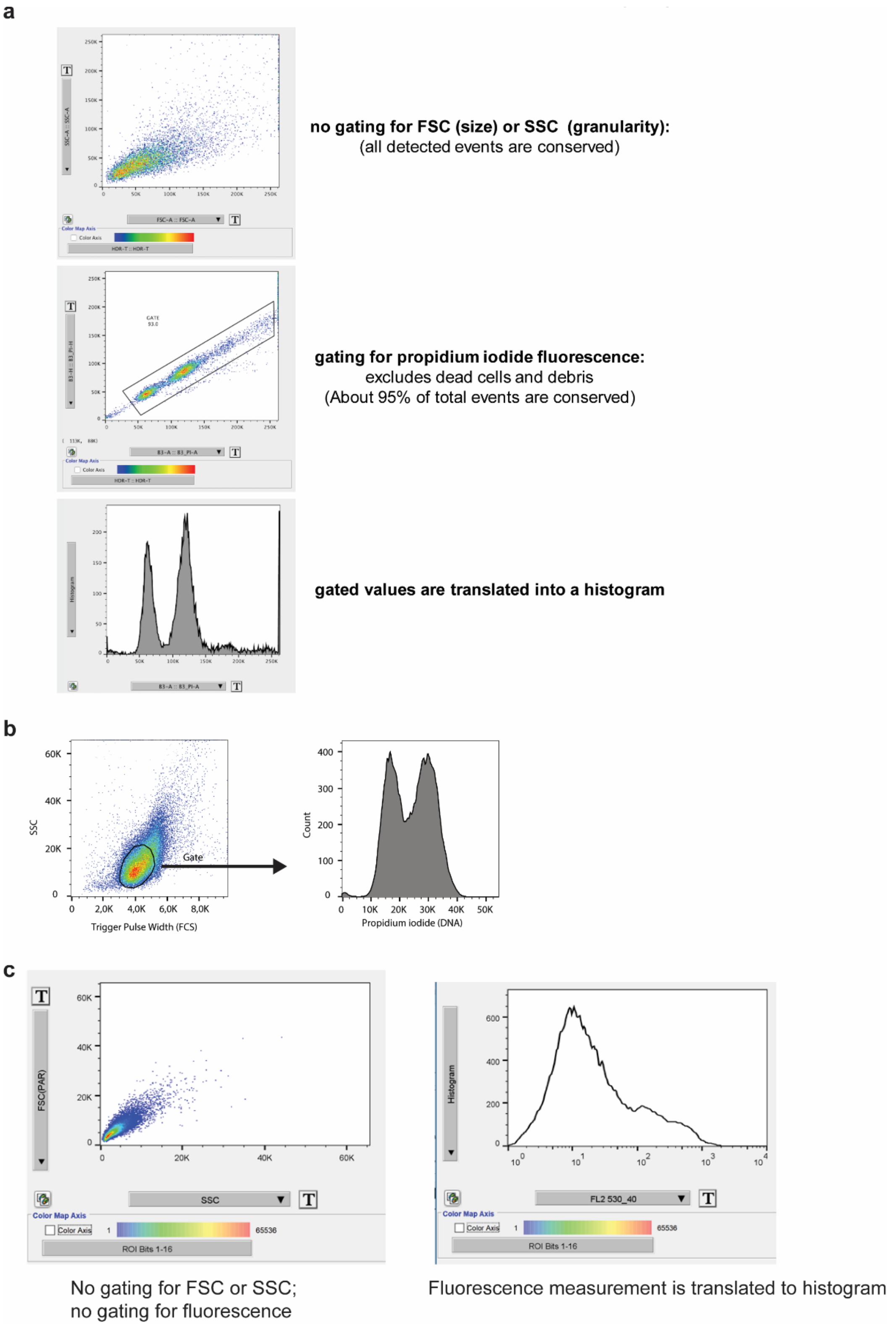

Supplementary Figure 10. FACS gating strategies

a. Gating strategy used for cell cycle progression analyses
b. Gating strategy used for cell cycle progression analyses in Supplementary Figure 7
c. Gating strategy used to measure ethanol-induced oxidation. In this case, no gating was performed, since the average fluorescence intensity at 530 nm is determined per sample. Screenshots shown are obtained using FlowJo software[13].

Supplementary Table 1. P-values

Figure	Comparison	P value
**Figure 8**	Pol2-myc-ARS305-39.5 kb 0 vs 6% 20 min	<0.0001
	Pol2-myc-ARS305-39.5 kb 0 vs 6% 40 min	0.0004
	Pol2-myc-ARS305-39.5 kb 0 vs 6% 60 min	0.0134
	Pol2-myc-ARS305-42.5 kb 0 vs 6% 40 min	0.0223
	Pol2-myc-ARS305-42.5 kb 0 vs 6% 80 min	0.0028
	Rev3-FLAG-ARS305-36.5 kb 0 vs 6% 60 min	0.0178
**Supp Figure 2c**	0% EtOH and 6% EtOH	$1.046\ 10^{-13}$
	6% EtOH and 1 mM fomepizole	0.01
	6% EtOH and 5 mM fomepizole	$1.301\ 10^{-4}$
**Supp Figure 2d**	0% EtOH and 6% EtOH	0.0019
**Supp Figure 4c**	RM11-1a 0 vs 0.001% acetaldehyde	$1.115\ 10^{-4}$
	RM11-1a 0 vs 0.01% acetaldehyde	$6.764\ 10^{-4}$
	RM11-1a 0 vs 0.1% acetaldehyde	$1.294\ 10^{-4}$
**Supp Figure 4d**	W303 0 vs 0.001% acetaldehyde	$7.075\ 10^{-5}$
	W303 0 vs 0.01% acetaldehyde	$1.714\ 10^{-5}$
**Supp Figure 4e**	YJM789 0 vs 0.001% acetaldehyde	$1.963\ 10^{-9}$
	YJM789 0 vs 0.01% acetaldehyde	0
	YJM789 0 vs 0.1% acetaldehyde	0

Supplementary Table 2. List of strains used in this study

**Name**	**Genotype**	**reference**
KV172	prototrophic haploid S288c, *MATa*	FY5 from[14]
VK111	KV172 *flo1Δ flo10Δ flo11Δ*	[15]
RM11-1a	*MAT***a** *leu2Δ0 ura3-Δ0 HO::kanMX*	[16]
ML904-14C	W303 Mat**a** *ADE2 trp1-1 LYS2 RAD5 CAN1*	[17]
VK3847	ML904-14C *SSD1*^{G2094C}	This study
EV14	VK111 *ADH2::KanMx*	This study
EV19	VK111 *TEF1-ADH2*	This study
KV173	prototrophic haploid S288c, *MAT**a***	FY4 from [13]
VK91	KV173 *ura3Δ flo1Δ flo10Δ flo11Δ*	This study
VK3703	VK91 pESC-VHL-mCherry	This study
W6986-1B	W303 *MATa ADE2 BAR1 trp1-1 LYS2 RAD5* YFP-8ala-Rnr3	[18]
IG101-12D	W303 *MAT**a** ADE2* YFP-8ala-Sml1 *RAD5* Rad52-yEmRFP *LYS2 trp1-1*	[19]
CC71-34B	W303 *MAT**a** ADE2 RAD5 LYS2 trp1-1* CMR1-yEmRFP MRC1-4Ala-YFP CFP-GGPGG-Pol30	This study
VK3761	*VK111 TEF1-MRC1*	This study
VK3831	VK111 *POL30* $^{A490C,\ A491G}$	This study
MVP1101	VK111 *REV3::HYG*	This study
MVP1105	VK111 *REV1::HYG*	This study
VK3608	VK111 *REV7::HYG*	This study
VK3614	VK111 *RAD30::HYG*	This study
VK3918	VK111 *YKU70::KanMx*	This study
ML996-2D	W303 *MAT**a** ADE2 trp1-1 LYS2 RAD5* Rev1-3x-FLAG::*HIS3MX* Rev3-3x-FLAG::*HIS3MX* Rev7-3x-FLAG::*HIS3MX* Pol2-13myc::*KanMx*	This study
VK3928	VK111 *PSO2::KanMx*	This study
PP2226	W303 *MAT**a** RAD5 ura3::URA3::GPD-*TK_7 *hENT1::AUR1c*	This study
BY4741	Auxotrophic haploid S288c, mat**a** BY4741 *his3Δ1 leu2Δ0 met15Δ0 ura3Δ0*	[14]
YJM789	Auxotrophic haploid YJM789	[20]

Supplementary Table 3. List of primers used in this study

Primer Name	Sequence
5314-KV-Yku70del_Fw	TGTTAAGTGACTCTAAGCCTGATTTTAAAACGGGAATATTCAGCTGA AGCTTCGTACGC
5315-KV-Yku70del_Rv	TTGTATGTAACGTTATAGATATGAAGGATTTCAATCGTCTCATAGGC CACTAGTGGATCTG
5316-KV-Yku70del_ck_Fw	GGATTGCTTTAAGGTAGCTACC
5317-KV-Yku70del_ck_Rv	CATCAAATACCCTACCCTACC
5248-KV-CAN1_Fw	TCTTCAGACTTCTTAACTCC
5249-KV-CAN1_Rv	ATAGTAAGCTCATTGATCCC
5250-KV-CAN1-seq1_Fw	GACGTACAAAGTTCCACTG
5251-KV-CAN1-seq2_Fw	TCAAAGAACAAGTTGGCTCC
5252-KV-CAN1-seq3_Rv	TAGATGTCTCCATGTAAGCC
5253-KV-CAN1-seq4_Rv	AACTTTGATGGAAGCGACCC
5280-guide RNA Pol30_1	GATCGTACAAACTTTATTGTTTCTTG
5282-repair template Pol30_1	TTGTCCCAATTGAGTGATTCTATTAATATCATGATCACCCGAGAAAC AATAAAGTTTGTA
5283-repair template Pol30_2	GTTTTATTATGACTGAACCTGATCCGATATCACCGTCAGCTACAAAC TTTATTGTTTC
5284-guide RNA Pol30_3	AAAACAAGAAACAATAAAGTTTGTAC
5261-KV-Pol30K164R_seq1	CTGTCATTGCCATCTTCCG
5262-KV-Pol30K164R_seq2	GGATGTTCCATATCCACGAAT
5220-KV-Rev7_del_Fw	CCAAGAAGAAAAAAAAAATAGTAATCGTTGCGTCAGCTTTCAGCTG AAGCTTCGTACGC
5221-KV-Rev7_del_Rv	ATTTAATTTTAATTCCATTCTTCAAATTTCATTTTTGCACCATAGGCCA CTAGTGGATCTG
5222-KV-Rev7_del_ck_Fw	CAGTCAATCGAAACCACTAGC
5223-KV-Rev7_del_ck_Rv	CATCCCAGAAATGCTGTGAT
5224-KV-Rad30_del_Fw	CTGCTCATTTTTGAACGGCTTTGATAAAACAAGACAAAGCCAGCTGA AGCTTCGTACGC
5225-KV-Rad30_del_Rv	TTTAGTTGCTGAAGCCATATAATTGTCTATTTGGAATAGGCATAGGC CACTAGTGGATCTG

5226-KV_Rad30_del_ck_Fw	CCGATCATAGGATACCTATTGTATG
5227-KV-Rad30_del_ck_Rv	GTTCTTCTTATCAACAAAACCTGG
5267-KV-Mrc1-TEF_Fw	AGACAAACAACTAAGGAAGTTCGTTATTCGCTTTTGAACTTATCACCAAATATTTTAGTGCAGCTGAAGCTTCGTACGC
5268-KV-Mrc1-TEF_Rv	GTAGGTGGTAGTTCTCTTCTTTGCAGTCAACGAGGACAAAGCATGCAAGGCATCATCCATTTTTCTAGAAAACTTAG
5269-KV-Mrc1-TEF_ck_Rv	GTCAACTCTGGCGGATTGTC
5244-KV-MRC1del_Fw	TCGTTATTCGCTTTTGAACTTATCACCAAATATTTTAGTGCAGCTGAAGCTTCGTACGC
5245-KV-MRC1del_Rv	CTGGAGTTCAATCAACTTCTTCGGAAAAGATAAAAAACCACATAGGCCACTAGTGGATCTG
5246-KV-MRC1del_ck_Fw	GTAAAACGCGTTTGCTTCAA
5247-KV-MRC1del_ck_Rv	CATGACTATGGCTTGGCCTA
4856-KV-ADH2del_Fw	ATATCAAGCTACAAAAAGCATACAATCAACTATCAACTATTAACTATATCGTAATACACACAGCTGAAGCTTCGTACGC
4857-KV-ADH2del_Rv	TACTAATATAGGCATACTTGATAATGAAAACTATAAATCGTAAAGACATAAGAGATCCGCCATAGGCCACTAGTGGATCTG
4858-KV-ADH2del_ck_Fw	ATATAAATAGAGTGCCAGTAGCG
4859-KV-ADH2del_ck_Rv	CGTGAAACTTCGAACACTGT
4981-KV-TEF1p-ADH2_Fw	ATATCAAGCTACAAAAAGCATACAATCAACTATCAACTATTAACTATATCGTAATACACACAGCTGAAGCTTCGTACGC
4982-KV-TEF1p-ADH2_Rv	CTCCAACTTGCCGTTGGATTCGTAGAAGATAATGGCTTTTTGAGTTTCTGGAATAGACATTTTTCTAGAAAACTTAG
MVP95_REV1del_Fw	GTAACGAGTTGACAGATTTTCTCAAAATAAATCGATACTGCATTTCTAGGCATATCCAGCGCAGCTGAAGCTTCGTACGC
MVP96_REV1del_Rv	ACAGGTAATGTTCGCAAACTGCGTGTTTACTGTATGCTGAAATGTTTTTTTTTTTTAATGCATAGGCCACTAGTGGATCTG
MVP97_REV1check_Fw	TGGCATAGTCTAAAGACCTG
MVP98_REV1check_Rv	TCAAGGATTCAAAGGAGGAG
MVP87_REV3del_Fw	AAGAGAAAGTATTTGAGTCAATACAAAACTACAAGTTGTGGCGAAATAAAATGTTTGGAACAGCTGAAGCTTCGTACGC
MVP88_REV3del_Rv	AACGTTATACATAGAAACAAATAACTACTCATCATTTTGCGAGACATATCTGTGTCTAGAGCATAGGCCACTAGTGGATCTG
MVP89_REV3check_Fw	CCAAGAATCCCTGTGGTC

MVP90_REV3check_Rv	TAGCAATCCACTCTTAGAGG
ACS305FW	TTTGGAGCTCAAGTGGATTGAG
ACS305RV	TGAAACTGGACATATTTGAGGAATTT
3053KBUFW	TGAAGACGCTGGAACGTTAGATT
3053KBURV	TGGACTTGGTGTTTGCAGATTG
3053KBDFW	TGCGAACGCTAAGGAGCTAGA
3053KBDRV	CAATGATGTGTCCCTAATCGTTATG
ACS501FW	AAGCAAATTGCAGAAGGTTATGAA
ACS501RV	TTCAAGGCTCTAGCATATGAAACG
REV1-S3-F	TTACCAGACTGTGCGTAAACTTGACATGGACTTTGAAGTTCGTACGC TGCAGGTCGAC
REV1-S2-R	GCGTGTTTACTGTATGCTGAAATGTTTTTTTTTTTTTAATATCGATGA ATTCGAGCTCG
REV3-S3-F	TGTTCAAAGGGAAGAAGCATTAATATCTCTAAATGATTGGCGTACG CTGCAGGTCGAC
REV3-S2-R	ATAACTACTCATCATTTTGCGAGACATATCTGTGTCTAGAATCGATG AATTCGAGCTCG
REV7-S3-F	TCAATATGAAGAGGGCGAGAGCATTTTTGGATCTTTGTTTCGTACGC TGCAGGTCGAC
REV7-S2-R	ATTTAATTTTAATTCCATTCTTCAAATTTCATTTTTGCACATCGATGAA TTCGAGCTCG
5288-KV-guide-SSD1-1	GATCGAGAATATAGGAGGAATTTTAG
5289-KV-guide-SSD1-2	AAAACGATATTCTACTCATTAGTGTC
5290-KV-repair_SSD1_1	AACCAAGTTTTCAGCCGCTACCATTAACGGCTGAAAGTCTAGAATAT AGGAGGAATTTTA
5291-KV-repair_SSD1_2	AGCTCGGAAATTGCAAAGATATTGTACTCATTAGTGTCCGTAAAATT CCTCCTATATTCT
5300-KV-SSD1_seq_Fw	TTCAGCCGCTACCATTAACG
5301-KV-SSD1_seq_Rv	CAATATGGCTGGTCACATCA
5352-KV-PSO2_del_Fw	AGCATACGCACTAGTGACTAATTTGGGTGGTCGGTTGATTCAGCTG AAGCTTCGTACGC
5353-KV-PSO2_del_Rv	TTTTTACCCCCTTTTCTTTTTTTTTGTTTTCCTTTTTTGTTCATAGGCCAC TAGTGGATCTG

5354-KV-PSO2_del_ck_Fw	TTTTGTCATGGAAACGGACTG
5355-KV-PSO2_del_ck_Rv	GATCAAACGTTGGACTCTGAG

Supplementary Table 4. List of plasmids used in this study

**Plasmid name**	**genotype**	**reference**
pUG6	*loxP-TEF-KanMX-TEF-loxP*	[21]
pCB1	*loxP-TEF-HYG-TEF-loxP*	[22]
KV2512	CEN, ARS, *loxP-KanMx-loxP-TEF1p*	[23]
pESC-mCherry-VHL	*AMP^R URA3 pGAL1-ESCmCherry-VHL*	[24]
pV1382	CEN, ARS, NatR, *TEF1-CaCas9 SNR52-sgRNA*	[25]
pBP81	3xFLAG::HIS3	Boris Pfander

Supplementary References

1. Trapnell, C. et al. Differential analysis of gene regulation at transcript resolution with RNA-seq. *Nat Biotechnol* **31**, 46-53 (2013).
2. Trapnell, C. et al. Differential gene and transcript expression analysis of RNA-seq experiments with TopHat and Cufflinks. *Nat Protoc* **7**, 562-578 (2012).
3. Kim, D. et al. TopHat2: accurate alignment of transcriptomes in the presence of insertions, deletions and gene fusions. *Genome Biol* **14**, R36 (2013).
4. Trapnell, C. et al. Transcript assembly and quantification by RNA-Seq reveals unannotated transcripts and isoform switching during cell differentiation. *Nat Biotechnol* **28**, 511-515 (2010).
5. Huang da, W., Sherman, B.T. & Lempicki, R.A. Systematic and integrative analysis of large gene lists using DAVID bioinformatics resources. *Nat Protoc* **4**, 44-57 (2009).
6. Huang da, W., Sherman, B.T. & Lempicki, R.A. Bioinformatics enrichment tools: paths toward the comprehensive functional analysis of large gene lists. *Nucleic acids research* **37**, 1-13 (2009).
7. Huang da, W. et al. Extracting biological meaning from large gene lists with DAVID. *Curr Protoc Bioinformatics* **Chapter 13**, Unit 13 11 (2009).
8. Eckert-Boulet, N., Rothstein, R. & Lisby, M. Cell biology of homologous recombination in yeast. *Methods Mol. Biol.* **745**, 523-536 (2011).
9. Heim, R. & Tsien, R.Y. Engineering green fluorescent protein for improved brightness, longer wavelengths and fluorescence resonance energy transfer. *Curr. Biol.* **6**, 178-182 (1996).
10. Ormo, M. et al. Crystal structure of the Aequorea victoria green fluorescent protein [see comments]. *Science* **273**, 1392-1395 (1996).
11. Keppler-Ross, S., Noffz, C. & Dean, N. A new purple fluorescent color marker for genetic studies in Saccharomyces cerevisiae and Candida albicans. *Genetics* **179**, 705-710 (2008).
12. Bianco, J.N. et al. Analysis of DNA replication profiles in budding yeast and mammalian cells using DNA combing. *Methods* **57**, 149-157 (2012).
13. FlowJo™ Software [software application] Version 10. Becton, Dickinson and Company; 2019.

14. Brachmann, C.B. et al. Designer deletion strains derived from Saccharomyces cerevisiae S288C: a useful set of strains and plasmids for PCR-mediated gene disruption and other applications. *Yeast* **14**, 115-132 (1998).
15. Voordeckers, K. et al. Adaptation to High Ethanol Reveals Complex Evolutionary Pathways. *PLoS Genet* **11**, e1005635 (2015).
16. Brem, R.B., Yvert, G., Clinton, R. & Kruglyak, L. Genetic dissection of transcriptional regulation in budding yeast. *Science* **296**, 752-755 (2002).
17. Silva, S. et al. Mte1 interacts with Mph1 and promotes crossover recombination and telomere maintenance. *Genes Dev* **30**, 700-717 (2016).
18. Andreson, B.L., Gupta, A., Georgieva, B.P. & Rothstein, R. The ribonucleotide reductase inhibitor, Sml1, is sequentially phosphorylated, ubiquitylated and degraded in response to DNA damage. *Nucleic acids research* **38**, 6490-6501 (2010).
19. Silva, S., Gallina, I., Eckert-Boulet, N. & Lisby, M. Live cell microscopy of DNA damage response in Saccharomyces cerevisiae. *Methods Mol Biol* **920**, 433-443 (2012).
20. Wei, W. et al. Genome sequencing and comparative analysis of Saccharomyces cerevisiae strain YJM789. *Proc Natl Acad Sci USA* **104**(31), 12825-30 (2007).
21. Guldener, U., Heck, S., Fielder, T., Beinhauer, J. & Hegemann, J.H. A new efficient gene disruption cassette for repeated use in budding yeast. *Nucleic acids research* **24**, 2519-2524 (1996).
22. Brown, C.A., Murray, A.W. & Verstrepen, K.J. Rapid expansion and functional divergence of subtelomeric gene families in yeasts. *Curr Biol* **20**, 895-903 (2010).
23. Nevoigt, E. et al. Engineering of promoter replacement cassettes for fine-tuning of gene expression in Saccharomyces cerevisiae. *Appl Environ Microbiol* **72**, 5266-5273 (2006).
24. Kaganovich, D., Kopito, R. & Frydman, J. Misfolded proteins partition between two distinct quality control compartments. *Nature* **454**, 1088-1095 (2008).
25. Vyas, V.K. et al. New CRISPR Mutagenesis Strategies Reveal Variation in Repair Mechanisms among Fungi. *mSphere* **3** (2018).
