# Supplementary material for: Ethanol exposure increases mutation rate through error-prone polymerases
Source: Nat Commun. 2020 Jul 21;11:3664. doi: 10.1038/s41467-020-17447-3 (PMC7374746; doi:10.1038/s41467-020-17447-3)
Supplement: Supplementary file 3 — Description of Additional Supplementary Files [file 41467_2020_17447_MOESM3_ESM.pdf]

Description of Additional Supplementary Files

File Name: Supplementary Data 1
Description: Mutations identified in can^R colonies after exposure to 0 or 6% ethanol.

File Name: Supplementary Data 2
Description: List of genes differentially expressed in ethanol. Tabs indicate the different comparisons between conditions, with T0 = start timepoint, E2 = 2 generations in ethanol, E4 = 4 generations in ethanol, G2 = 2 generations in glucose, G4 = 4 generations in glucose. Different headers represent gene name, locus, value 1 (transcript abundance value in condition 1), value 2 (transcript abundance value in condition 2), log2(fold_change), p-value (uncorrected pvalue), Q-value (FDR-adjusted p-value; using Benjamini-Hochberg correction for multiple-testing).

File Name: Supplementary Data 3
Description: Clusters of terms enriched in differentially expressed genes. Tabs indicate the different comparisons between conditions, with T0 = start timepoint, E2 = 2 generations in ethanol, E4 = 4 generations in ethanol, G2 = 2 generations in glucose, G4 = 4 generations in glucose.
