# Supplementary material for: Ethanol exposure increases mutation rate through error-prone polymerases
Source: Nat Commun. 2020 Jul 21;11:3664. doi: 10.1038/s41467-020-17447-3 (PMC7374746; doi:10.1038/s41467-020-17447-3)
Supplement: Supplementary file 7 — Reporting Summary [file 41467_2020_17447_MOESM7_ESM.pdf]

nature research

Corresponding author(s): Verstrepen Kevin and Lisby Michael

Last updated by author(s): 2020/05/21

Reporting Summary

Nature Research wishes to improve the reproducibility of the work that we publish. This form provides structure for consistency and transparency in reporting. For further information on Nature Research policies, see Authors & Referees and the Editorial Policy Checklist.

Statistics

For all statistical analyses, confirm that the following items are present in the figure legend, table legend, main text, or Methods section.

n/a	Confirmed	
☐	☒	The exact sample size (n) for each experimental group/condition, given as a discrete number and unit of measurement
☐	☒	A statement on whether measurements were taken from distinct samples or whether the same sample was measured repeatedly
☐	☒	The statistical test(s) used AND whether they are one- or two-sided<br>*Only common tests should be described solely by name; describe more complex techniques in the Methods section.*
☒	☐	A description of all covariates tested
☒	☐	A description of any assumptions or corrections, such as tests of normality and adjustment for multiple comparisons
☐	☒	A full description of the statistical parameters including central tendency (e.g. means) or other basic estimates (e.g. regression coefficient) AND variation (e.g. standard deviation) or associated estimates of uncertainty (e.g. confidence intervals)
☐	☒	For null hypothesis testing, the test statistic (e.g. F, t, r) with confidence intervals, effect sizes, degrees of freedom and P value noted<br>*Give P values as exact values whenever suitable.*
☒	☐	For Bayesian analysis, information on the choice of priors and Markov chain Monte Carlo settings
☒	☐	For hierarchical and complex designs, identification of the appropriate level for tests and full reporting of outcomes
☒	☐	Estimates of effect sizes (e.g. Cohen's d, Pearson's r), indicating how they were calculated

Our web collection on statistics for biologists contains articles on many of the points above.

Software and code

Policy information about availability of computer code

Data collection

No software was used for data collection.

Data analysis

All software used for data analysis is described in the manuscript. More specifically,
- fluctuation data analysis: R package RSalvador v1.7
- identification of mutations in CAN1 ORF: EMBOSS seqret version 6.6.0; FASTX-Toolkit version 0.0.14; MUSCLE version 3.8.31; AliView version 1.18.1
- RNA sequencing analysis: Tophat aligner v2.0.13; cufflinks v2.2.1; cuffdiff v2.2.1; DAVID 6.7
- cell synchronization data analysis: FlowJo v10
- data analysis: GraphPad Prism 7
- Images acquisition: softWoRx software version 7.0.0 (Applied Precision, Inc) software
- Fluorescence intensities of microscopy images: Volocity software version 5.4 (PerkinElmer).

For manuscripts utilizing custom algorithms or software that are central to the research but not yet described in published literature, software must be made available to editors/reviewers. We strongly encourage code deposition in a community repository (e.g. GitHub). See the Nature Research guidelines for submitting code & software for further information.

Data

Policy information about availability of data

All manuscripts must include a data availability statement. This statement should provide the following information, where applicable:

- Accession codes, unique identifiers, or web links for publicly available datasets
- A list of figures that have associated raw data
- A description of any restrictions on data availability

TThe RNA sequencing dataset generated and analysed during the current study has been deposited in Sequence Read Archive, as Bioproject PRJNA632734 (http://www.ncbi.nlm.nih.gov/bioproject/632734). The CAN1 sequencing data generated and analysed during the current study has been deposited at GenBank, accession

codes MT509124 - MT509357. Other data supporting the findings of this study are available in the associated Source Data File. More specifically, the following main figures have associated raw data: Figure 1, Figure 2, Figure 3 (b, c), Figure 4 (a, b), Figure 5 (a, b, d), Figure 6 (b, c), Figure 7 (a, b), Figure 8. The source data file also contains raw data for the following Supplementary Figures: Supplementary Figure 1, Supplementary Figure 2 (a-d), Supplementary Figure 3 (a,b), Supplementary Figure 4, Supplementary Figure 5, Supplementary Figure 6, Supplementary Figure 7, Supplementary Figure 8. The source data files underlying these figures are provided as Source Data File.

Field-specific reporting

Please select the one below that is the best fit for your research. If you are not sure, read the appropriate sections before making your selection.

☒ Life sciences ☐ Behavioural & social sciences ☐ Ecological, evolutionary & environmental sciences

For a reference copy of the document with all sections, see nature.com/documents/nr-reporting-summary-flat.pdf

Life sciences study design

All studies must disclose on these points even when the disclosure is negative.

Sample size

No sample size calculation was performed; sample size was determined based on previous studies. Sample sizes were chosen to be minimally n=3 (unless specified otherwise in the figure legend), the minimal to allow for statistical analysis and to ensure reproducibility.

Specifically for the mutation rate data (fluctuation assays): mutation rates are obtained using the golden standard approach, namely using the MSS-maximum likelihood method to estimate m (number of mutations) based on results obtained from many parallel cultures (in our case, at least 54 cultures were used per condition). Typical sample sizes used in fluctuation assays range from 18 to 72, see for example https://doi.org/10.1093/nar/gky751
and https://doi.org/10.1007/978-1-4939-7306-4_3

Specifically for Mrc1/INQ foci in Fig 6: At least 200 cells were analyzed per condition. The number of cells correspond to 8 fields on a microscope slide for each time point, which is the number of fields that can be acquired in less than 5 min. The limit of 5 min was chosen to avoid local depletion of nutrients on the slide during acquisition.

Specifically for ChIP analysis in Fig 8: Values are average of 3 biological replicates, with 2 technical repeats each (standard in the field). Sample size were 50 mL per time point.

Specifically for Rfa1 speckles in Fig S7: 187-847 cells for each time point and two biological replicates. The number of cells correspond to 8 fields on a microscope slide for each time point, which is the number of fields that can be acquired in less than 5 min. The limit of 5 min was chosen to avoid local depletion of nutrients on the slide during acquisition.

Data exclusions

No data was excluded.

Replication

To verify reproducibility of experiments, experiments were performed using biological replicates. Experiments were performed using 3 biological replicates, unless specified otherwise in the corresponding figure legend. Experiments presented good consistency. Specifically for the mutation rate measurements, there was experiment-to-experiment variation in the absolute mutation rate (as is always the case when performing fluctuation assays); but the fold changes in mutation rates were consistent between experiments.
In each of the fluctuation experiments described in this manuscript, mutation rates are always compared within one experiment. While there is experiment-to-experiment variation in the absolute mutation rate (as is always the case when performing fluctuation assays), the fold changes in mutation rate were always comparable - ranging from 2.6 to 3.5 fold increase when comparing 6% ethanol to 0% ethanol conditions for S288c WT strain.

Randomization

There was no need for sample randomization, we worked with samples if yeast cells.

Blinding

For analysis of colony counts of fluctuation assay data, investigators were blinded. For analysis of sequencing data (identification of mutations in CAN1 ORF), investigators were blinded. For Mrc1/INQ foci in Fig 6: The samples were not blinded because the distinct nature of Mrc1/INQ foci leaves little room for subjectivity during counting.
For the ChIP analysis in Fig 8: The samples were not blinded because the Ct values are calculates automatically by the qPCR programme, leaving no room for subjectivity.
For the Rfa1 speckles in Fig S7: The samples were not blinded because the degree of speckles were calculates automatically in the Volocity programme, leaving no room for subjectivity.
For other experiments not mentioned above: Investigators were not blinded, but viability measurements, flow cytometry studies and acetaldehyde measurements were performed independently by different investigators who were not aware of the results obtained by the others before putting all the data together.

Reporting for specific materials, systems and methods

We require information from authors about some types of materials, experimental systems and methods used in many studies. Here, indicate whether each material, system or method listed is relevant to your study. If you are not sure if a list item applies to your research, read the appropriate section before selecting a response.

Materials & experimental systems

n/a	Involved in the study
☐	☒ Antibodies
☒	☐ Eukaryotic cell lines
☒	☐ Palaeontology
☒	☐ Animals and other organisms
☒	☐ Human research participants
☒	☐ Clinical data

Methods

n/a	Involved in the study
☒	☐ ChIP-seq
☐	☒ Flow cytometry
☒	☐ MRI-based neuroimaging

Antibodies

Antibodies used	Antibodies used are mouse anti-ssDNA (DSHB; http://dshb.biology.uiowa.edu/autoimmune-ssDNA; autoanti-ssDNA was deposited to the DSHB by Voss, E.W. (DSHB Hybridoma Product autoanti-ssDNA)), rabbit anti-Rad53 antibody (kind gift of Dr. C. Santocanale, The gifted rabbit anti-Rad53 antibody is home-made from Corrado Santocanale (NUI, Galway). This antibody has been raised against the purified full length Rad53 protein expressed in Escherichia coli.), goat anti-mouse coupled to Alexa Fluor 647 (Catalog # A-21241, ThermoFisher), mouse monoclonal anti-Myc antibody (sc-40, 9E10, Santa Cruz Biotechnology), and mouse anti-FLAG (F1804, Sigma-Aldrich). Specific dilutions used are mentioned in the manuscript.
Validation	All antibodies are widely used. Examples of references for the mouse anti-ssDNA include Mol. Immunol. 19, 963-971.; Proteins 11(3), 159-175 and for the rabbit anti-Rad53 antibody include https://doi.org/10.15252/embj.201899319.<br>The following antibodies have a validation statement on the manufacturer's website: goat anti-mouse coupled to Alexa Fluor 647, anti-myc antibody and anti-FLAG antibody. For anti-myc antibody, statement reads: Widely used in combination with eukaryotic expression vectors encoding proteins with c-Myc (amino acids 408-439) epitope tag; for mouse anti-FLAG antibody, statement reads: For highly sensitive and specific detection of FLAG fusion proteins by immunoblotting, immunoprecipitation, immunohistochemisty, immunofluorescence and immunocyotchemistry.<br>Additionally, anti-myc antibody has been used in ChIP experiments, see doi: 10.1038/ncomms7533. Moreover, as a control in the ChIP experiment, this study also used beads without antibody added.

Flow Cytometry

Plots

Confirm that:

☒ The axis labels state the marker and fluorochrome used (e.g. CD4-FITC).

☒ The axis scales are clearly visible. Include numbers along axes only for bottom left plot of group (a 'group' is an analysis of identical markers).

☐ All plots are contour plots with outliers or pseudocolor plots.

☒ A numerical value for number of cells or percentage (with statistics) is provided.

Methodology

Sample preparation	Samples analysed were yeast samples. For synchronization experiments, cells were grown at 25°C in Synthetic Complete (SC) medium supplemented with 2% glucose and synchronized in G1 using 8 µg/ml α-factor (Biotem, France) for 150 min. Cells were released in S-Phase from the G1 arrest by α-factor degradation using 75 µg/ml Pronase and 20mM phosphate buffer. Samples from the time course experiments were processed with standard methods (cooling on ice after collection; and staining with propidium iodide) for subsequent flow cytometry analysis using a MACSQuant Analyze. For in vivo ROS measurements using H2DCFDA,<br>cells were grown as for standard fluctuation assay. H2DCFDA (Sigma, stock concentration: 5 mg/mL in DMSO) was added to each culture to a final concentration of 12.5 µg/mL and light-protected samples were incubated for 30 min at 30°C. Samples were divided over three Eppendorf tubes and incubated with nothing, 6% ethanol (final concentration) or 100 mM H2O2 for 2h at 30°C. Fluorescence was subsequently analysed using AttuneTM NxT Acoustic Focusing cytometer. A total of 50 000 cells was analysed per sample, and 3 independent cultures were analyzed for each treatment.
Instrument	For in vivo ROS measurements using H2DCFDA,, an AttuneTM NxT Acoustic Focusing cytometer was used. For cell cycle progression analysis, data were acquired on a MACSQuant Analyser (Miltenyi Biotec)
Software	FlowJo version 10
Cell population abundance	Populations were not sorted.
Gating strategy	For analysis of HCDFDA fluorescence: no gating was applied (mean fluorescence was analysed).<br>For cell cycle progression analysis: FSC/SSC; PI fluorescence.

See Supplementary Figure 10 for a figure exemplifying the gating strategies used.

☒ Tick this box to confirm that a figure exemplifying the gating strategy is provided in the Supplementary Information.
